# Sudden cardiac death among workers: a systematic review and meta-analysis

**DOI:** 10.1186/s13643-024-02504-5

**Published:** 2024-03-09

**Authors:** Carlotta Amantea, Enrico Pilia, Maria Francesca Rossi, Valerio Flavio Corona, Giuseppe Amato, Corrado Ciccu, Filippo Gavi, Paolo Emilio Santoro, Ivan Borrelli, Walter Ricciardi, Maria Rosaria Gualano, Umberto Moscato

**Affiliations:** 1 Department of Life Science and Public Health, Università Cattolica del Sacro Cuore, Largo Francesco Vito 1, Rome, 00168 Italy; 2 Department of Medical Sciences and Public Health, Section of Legal Medicine, University of Cagliari, Cagliari, Italy; 3 Department of Life Sciences and Public Health, Section of Hygiene, Università Cattolica del Sacro Cuore, Largo Francesco Vito 1, Rome, 00168 Italy; 4 Department of Urology, Fondazione Policlinico Universitario Agostino Gemelli IRCCS, Largo Francesco Vito 1, Rome, 00168 Italy; 5 Department of Women, Children and Public Health Sciences, Fondazione Policlinico Universitario Agostino Gemelli IRCCS, Largo Francesco Vito 1, Rome, 00168 Italy; 6 Saint Camillus International University of Health Sciences, UniCamillus, Via Sant’Alessandro 8, Rome, 00131 Italy

**Keywords:** Sudden cardiac death, Workers, Occupational health, Public health, Cardiovascular diseases

## Abstract

**Objective:**

Sudden cardiac death (SCD) is a rare and yet unexplained condition. The most frequent cause is myocardial infarction, while a small proportion is due to arrhythmogenic syndromes (e.g., channelopathies). This systematic review and meta-analysis aimed to provide a comprehensive overview of the prevalence and risk factors associated with SCD in workers.

**Material and methods:**

A search for eligible studies was performed utilizing three databases (PubMed, ISI Web of Knowledge, and Scopus). The inclusion criteria were fulfilled if sudden cardiac death due to channelopathy in workers was mentioned.

**Results:**

Out of the 1408 articles found across three databases, 6 articles were included in the systematic review but the meta-analysis was conducted on 3 studies The total sample included was 23,450 participants. The pooled prevalence of channelopathies in employees was 0.3% (95% CI 0.07–0.43%), of sudden cardiac death in employees was 2.8% (95% CI 0.37–5.20%), and of sudden cardiac death in employees with a diagnosis of cardiac channelopathies was 0.2% (95% CI 0.02– 0.30%).

**Conclusions:**

SCD is a serious and potentially preventable condition that can occur among workers. By identifying and addressing work-related risk factors, providing appropriate screening and interventions, and promoting healthy lifestyle behaviors, we can work to reduce the incidence of SCD and improve the cardiovascular health and well-being of workers.

## Introduction

The term “sudden death,” which was originally used more than 2400 years ago [1], refers to the passing away of an apparently healthy individual within an hour of the onset of symptoms or without witnesses within the previous 24 h. Most sudden deaths are cardiac in nature, other causes are neurological, e.g., epilepsy or intracranial hemorrhage, or pulmonary, e.g., pulmonary embolism [2]. The most frequent cause of sudden cardiac death is myocardial infarction in about 75% of cases, while arrhythmogenic syndromes account for about 20% and mainly affect individuals under 35 years of age. The remaining causes are due to congenital structural heart abnormalities and occur in childhood [3]. Arrhythmogenic syndromes are classified into two large groups: primary cardiomyopathies (15%) and channelopathies (5%) [4]. The former is usually identifiable at autopsy in three main features: hypertrophic, dilated, and arrhythmogenic. In the early stages of cardiomyopathies, the heart may be normal, and alterations may be only detectable through an electron microscope [5]. However, even in the presence of a macroscopic alteration, it may be difficult to distinguish between a benign and a pathological condition. The latter is represented by pathology associated with ion channel alterations, i.e., channelopathies. These are congenital defects associated with ion channel protein abnormalities, related to electrical disorders affecting the genesis and conduction of the electrical impulse [4]. The most common channelopathies are long QT syndrome (LQTS) and Brugada syndrome (SB) [6, 7], which can be detected using a resting electrocardiogram (ECG) as part of worker health surveillance visits. However, there are other channelopathies such as short QT syndrome (SQTS), catecholaminergic polymorphic ventricular tachycardia (CPVT), arrhythmogenic right ventricular cardiomyopathy (ARVC), and other hereditary heart rhythm disorders [8].

LQTS is a group of inherited disorders characterized by dysfunction of myocardial electrolyte channels leading to a delay in ventricular repolarisation, which is visible on the ECG as prolongation of the QT segment. A prolonged QT interval of > 440 ms in men and > 460 ms in women is considered abnormal [9].

Brugada syndrome (SB) [10] is a hereditary channelopathy with autosomal dominant transmission. It presents with an electrocardiographic pattern characterized by ST-segment elevation and positive T wave with 'saddleback' morphology in the right precordial leads (V1 to V3) or 'convex or domed' ST-segment elevation with negative T [11]. Other ECG changes such as negative T waves or changes similar to a right bundle branch block may be present. The above-described alterations may be absent or minimal and could be slatentized or accentuated by sodium channel blocking drugs, especially Class IC antiarrhythmics (Flecainide), or by an exercise test [12].

Sudden cardiac death (SCD) is a leading cause of mortality worldwide, and it remains a major public health concern [13]. According to the Centers for Disease Control and Prevention (CDC), SCD accounts for approximately 10–15% of all deaths in the USA each year. However, there is limited data available specifically on the prevalence of SCD among workers [14] While SCD can affect anyone, certain populations may be at a higher risk, including individuals in the workforce. Work-related stress, sedentary lifestyles, and other occupational hazards can contribute to an increased risk of SCD in employees [15].

This systematic review and meta-analysis aimed to provide a comprehensive overview of the prevalence and risk factors associated with SCD in employees. Overall, our study emphasizes the importance of identifying sudden cardiac death as a possible occupational hazard and the need for more investigation and action to prevent these tragic events in the workplace.

## Methods

The research was conducted between October 2022 and October 2023, and in accordance with the Preferred Reporting Items for Systematic Reviews and Meta-Analyses [16], a systematic review was conducted. The PRISMA guidelines suggest that the search is performed across multiple databases; therefore, we have chosen three databases due to their relevance in the medical and biomedical fields and due to their bibliometric characteristics: PubMed, ISI Web of Knowledge, and Scopus [17]. Documents written in the English language were reviewed (the English language filter was applied to the search), and no time restrictions were imposed as a filter (meaning all available scientific literature was included independently of how long ago it was published). The PECO approach was used to design the search query, using workers as the study population (P), affected by channelopathy or cardiac disease (E, exposure), and who died of sudden cardiac death (O, outcome); control (C) was not identified due to the topic of the review.

- The following query was employed for the bibliographic search:
- [(“cardiac” OR “heart”).
- AND
- (“arrest” OR “attack” OR “arythmia” OR (“sudden” AND “death”)).
- AND (channelopath* OR canalopat* OR “long QT syndrome” OR “short QT syndrome” OR “brugada syndrome”).
- AND (occupational OR work* OR employ*)].

Duplicates were eliminated after obtaining the articles from all the chosen databases, and the initial screening of the articles by title and abstract was carried out using the website tool Rayyan [18]. This allowed the researchers to independently screen the articles in accordance with the triple-blind methodology to lessen selection bias.

### Inclusion criteria

The search was limited to articles written in the English language, and no time restrictions were set. The inclusion criteria were fulfilled if sudden cardiac death due to channelopathy in workers was mentioned.

### Exclusion criteria

Articles that did not mention sudden cardiac death, channelopathies, or sudden cardiac death from cardiac structural alterations were excluded. Entries were also excluded if the sudden cardiac death was caused by other biological factors (myocardial infarction, congenital or acquired cardiomyopathies). Exclusion criteria also included the absence of the full text or articles inconsistent with the purpose of the research (conference proceedings, vignettes, etc.).

### Data extraction and synthesis

Data from the included articles were extracted by three researchers (E.P., C.A., and C.C.) and reported the results in an Excel sheet. One author (E.P.) extracted the data from the included research, while two other authors (C.A. and C.C.) validated the extracted data. A fourth author (V.F.C.) intervened when irreversible disputes arose between the first three authors. The information to be collected was pre-determined and included: author; year and country in which the research was conducted; study design; sample size; number of affected; average age; class of worker; possible follow-up; deaths as a result of channelopathies; type of channelopathies; possible cardiovascular risks.

### Quality assessment

The Newcastle–Ottawa Scale was used to evaluate the methodological quality of each of the included studies [19].

### Statistical analysis

By carrying out a meta-analysis of proportion [20], the pooled prevalence of sudden cardiac death among workers was assessed. The I2 statistic was used to evaluate the heterogeneity of the research. 25%, 50%, and 75% were judged low, moderate, and high levels of heterogeneity, respectively. Due to the high level of heterogeneity, a random-effects model was used for meta-analysis. Using the Egger regression model, publication bias was evaluated. As a significant level, a *p* value < 0.05 was chosen. A forest plot was used to demonstrate the pooled prevalence. STATA Software/MP, Version 14.1 (StataCorporation, College Station, TX, USA) and RevMan5, were used for the statistical analyses.

## Results

**Fig. 1:**
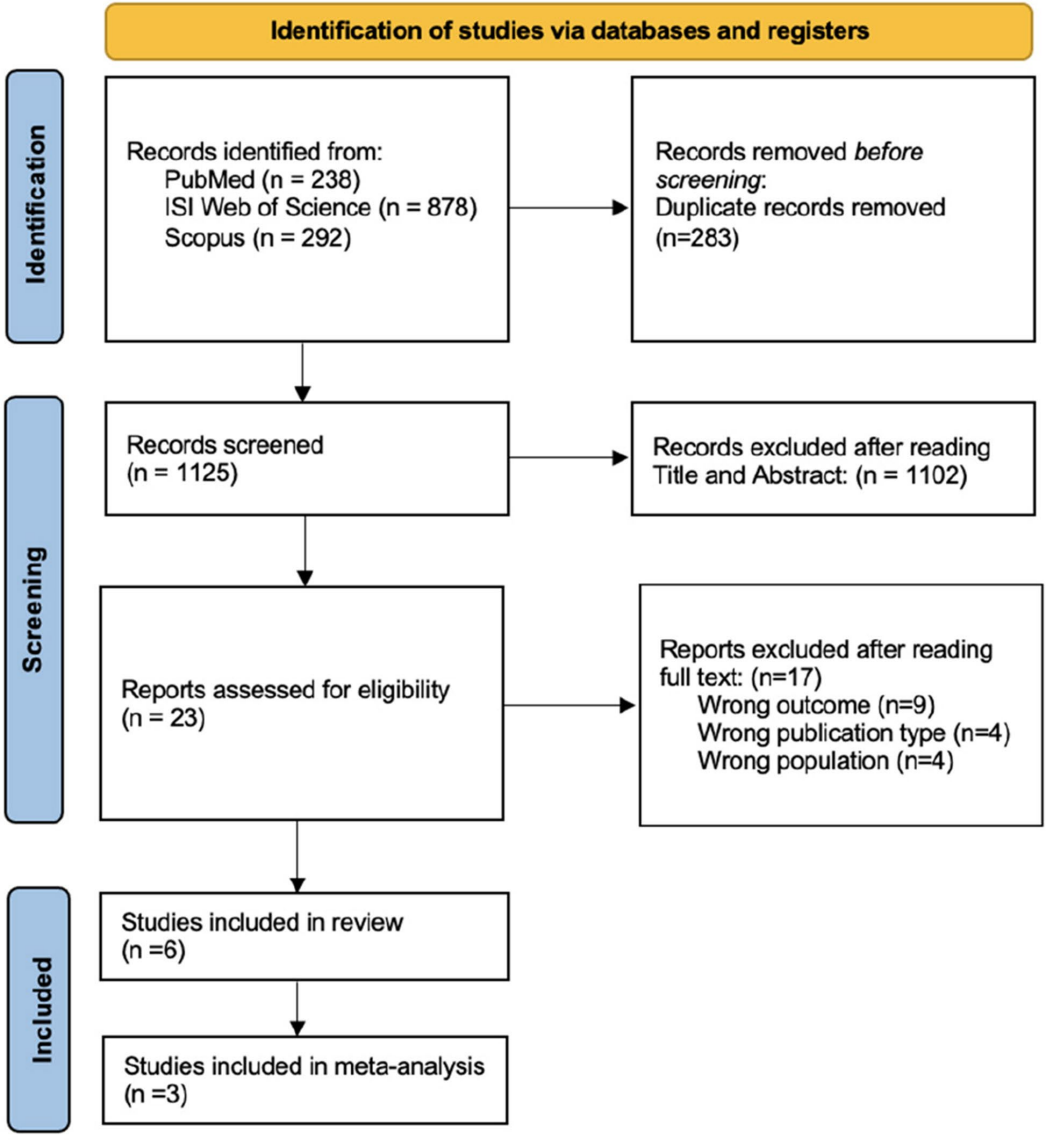
PRISMA flowchart for study selection

The initial search resulted in 1408 relevant studies among all three databases (238 Pubmed, 878 Web of Science, and 292 Scopus). After duplicate removal (283 duplicate articles), the initial search resulted in 1125 eligible papers. With the exclusion of 1102 papers after screening the abstracts, full texts of 23 articles were reviewed and 17 were excluded by full text. Subsequently, 6 articles were included in the systematic review and meta-analysis. Figure 1 shows the complete article selection process. Out of the 6 studies included in the review, 2 (25%) were conducted in the United States [13, 21], 1 (12.5%) in Italy [22], 1 (25%) in Denmark [23], 1 (25%) in China [24], and 1 (12.5%) in Germany [25]. Concerning study design, all 6 studies were retrospective studies. The main results are summarized in Table 1.

**Table 1:** Main characteristics of the included studies

Author	Year	Country	Study design	Sample size	Total SCD	CH^*^	Exitus by CH^*^	Mean age (SD)	Type of worker
Maron et al. [21]	2003	USA	Retrospective study	584	286	3	3	17 ± 3	Competitive athletes
Gallagher et al. [22]	2008	Italy	Retrospective study	12012	1	31	1	29.9 ± 9	N.R.**
Winkel et al. [23]	2012	Denmark	Retrospective study	6332	470	25	25	29 (IQR:22–33)	N.R.**
Zhenglian et al. [24]	2016	China	Retrospective study	5106	49	3	3	32.6 ± 10.3	Farmers, migrate workers, prisoners, researchers policewoman, soldiers, and railway
Hellenthal et al [25]	2017	Germany	Retrospective study	200	60	9	9	30.6 ± 7.3	Truck driver
Peterson et al [13]	2020	USA	Retrospective study	179	105	17	17	16.6 (Range: 11–29)	Competitive athletes

^*^CH Channelopathies

^**^N.R. Not reported

**Fig. 2:**
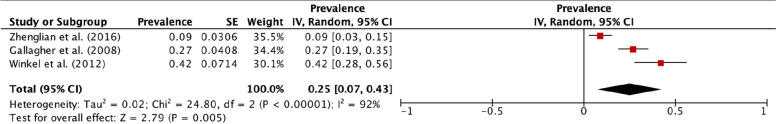
Forest plot: pooled prevalence of cardiac channelopathies in the study population

**Fig. 3:**
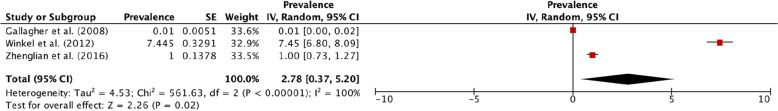
Forest plot: pooled prevalence of sudden cardiac death of the eligible studies

**Fig. 4:**
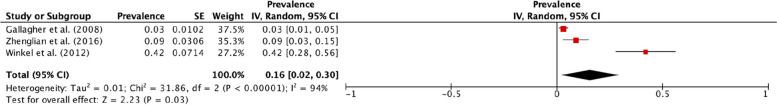
Forest plot: pooled prevalence of sudden cardiac death in workers with channelopathies of the eligible studies

**Fig. 5:**
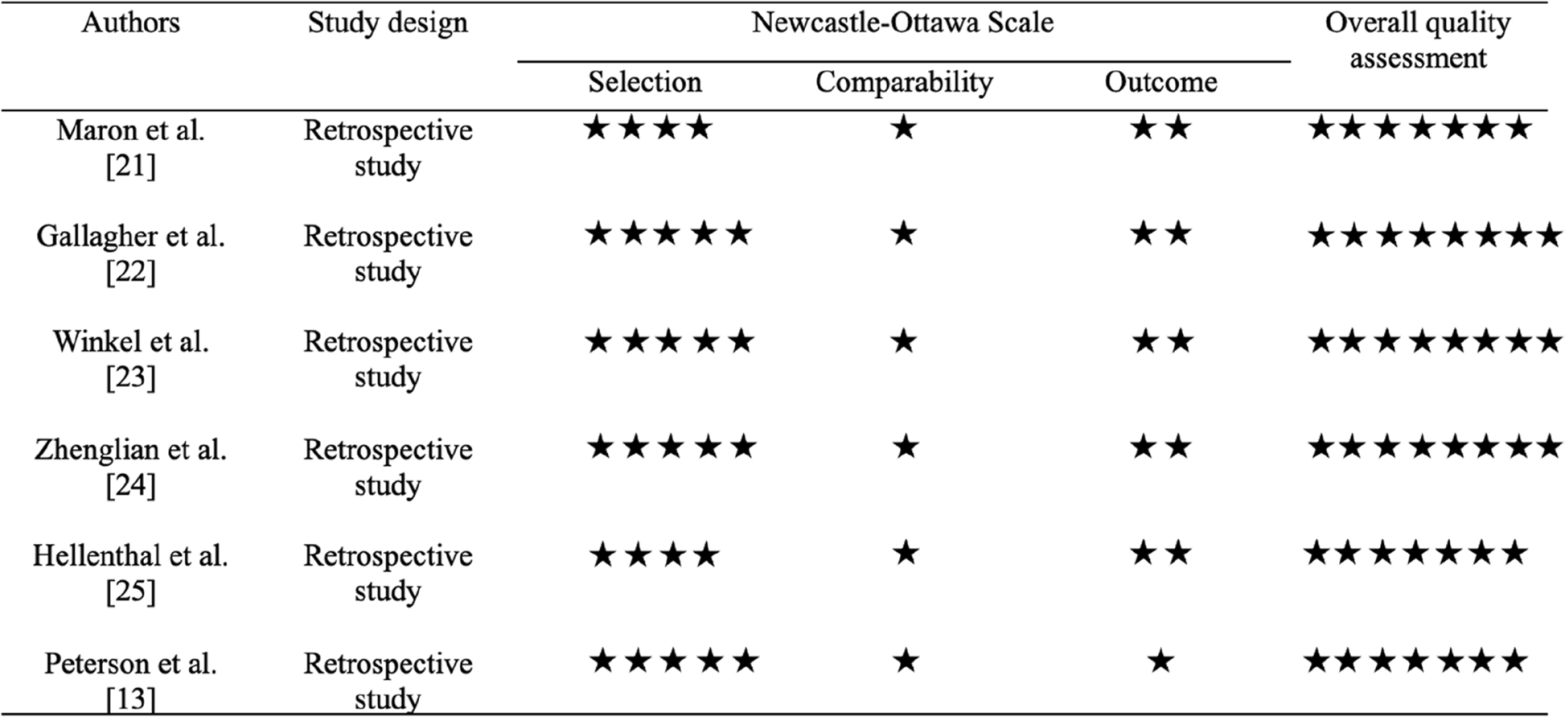
Quality assessment of included studies through the Newcastle–Ottawa Scale

A proportional meta-analysis was carried out on the included studies, with the aim of investigating the pooled prevalence of cardiac channelopathies, sudden cardiac death in employees, and sudden cardiac death in employees with a diagnosis of cardiac channelopathies. After sensitivity analysis 3 studies were excluded due to the high heterogeneity [13, 21, 25]. Therefore, the meta-analysis was conducted on 3 studies [22–24]The total sample included was 23,450 participants. The pooled prevalence of channelopathies in employees was 0.3% (95% CI 0.07–0.43%) (Fig. 2), the pooled prevalence of sudden cardiac death in employees was 2.8% (95% CI 0.37–5.20%) (Fig. 3), and the pooled prevalence of sudden cardiac death in employees with a diagnosis of cardiac channelopathies was 0.2% (95% CI 0.02–0.30%) (Fig. 4). All studies had a low risk of bias assessed by the Newcastle–Ottawa Scale, and all were assessed to have at least an adequate methodological quality (score ≥ 6) (Fig. 5). Heterogeneity was *I*^2^ = 92% (*p* < 0.001), for the pooled prevalence of channelopathies among all individuals. No significant publication bias was found (*p* > 0.5). Heterogeneity was *I*^2^ = 100% and 94% (*p* < 0.001), for the pooled prevalence of sudden cardiac death among all individuals and for the pooled prevalence of sudden cardiac death in workers with channelopathies, respectively. No significant publication bias was found (*p* > 0.5). A random effects model was used due to the high heterogeneity.

## Discussion

The purpose of this systematic review and meta-analysis was to present a comprehensive overview of the prevalence and risk factors for sudden cardiac death (SCD) in workers. Our findings suggest that the pooled prevalence of SCD in employees was 2.8%, channelopathy was 0.3%, and sudden cardiac death due to channelopathy was 0.2%.

The prevalence of SCD among workers can vary depending on many factors, including age, gender, occupation, and underlying health conditions. Generally, workers who are exposed to physical or mental stress, have sedentary lifestyles or have pre-existing heart conditions may be at higher risk for SCD.

A study published in the European Heart Journal in 2019 found that the risk of SCD was higher among blue-collar workers compared to white-collar workers. The study also found that workers with low socioeconomic status had a higher risk of SCD compared to those with high socioeconomic status [26]. It is important to note that SCD can occur in anyone, regardless of occupation or socioeconomic status. The study’s finding that workers with low socioeconomic status have a higher risk of SCD suggests that factors such as access to healthcare, lifestyle, and stress levels related to economic stability play a significant role. It's crucial to understand that SCD can occur in anyone, irrespective of their occupation or socioeconomic status. However, recognizing the roles of occupational stress, lifestyle, and health disparities is important in addressing and mitigating these risks. Workplace wellness programs and health screenings can be beneficial in identifying at-risk individuals and promoting heart-healthy practices.

Our study also showed that competitive athletes present earlier manifestations of the disease than other workers. This may be justified precisely by the fact that the former starts their activity much earlier.

The average age of onset of SCD due to channelopathies in competitive athletes was 17 ± 3 years [21] and 16.6 years [13], whereas in other categories of workers, Table 1 showed a higher average age. We do not know whether this phenomenon is really applied to the general population, what we hypothesize is an anticipation of the phenomenon as athletes are in excellent shape and have peak performance at a young age, while workers in other sectors are also prone to these sudden cardiac deaths because they are exposed to increasing cardiovascular risks over time, for example, farmers 44.9% and migrant workers 22.4% [24]. It was not possible to ascertain whether there is a confounding bias in the diagnosis process when it comes to competitive athletes, who receive an early diagnosis of channelopathies due to close and frequent cardiovascular check-ups from an early age, and other workers, who receive a later diagnosis due to later cardiological screening during the health surveillance visit. In relation to mortality, on the other hand, it is not possible to define whether athletes actually suffer early on from SCD due to their competitive sporting activity, which would therefore play a triggering role, as opposed to workers in other sections who are exposed to increasing cardiovascular risks over time.

One notable observation is that there is no increased occurrence of sudden cardiac death (SCD) caused by channelopathies in a particular occupation among the individuals examined. SCD is a devastating event that can occur in apparently healthy individuals, often with little or no warning. While the exact causes of SCD are not always clear, there is evidence to suggest that certain work-related risk factors may contribute to an increased risk of SCD among workers [27–29]. In this discussion, we will explore some of these risk factors and strategies to prevent SCD among workers. Long working hours, shift work [30], job strain [31], and work-related stress [32] have all been linked to an increased risk of cardiovascular disease, including SCD. Long working hours and shift work, in particular, have been associated with disruptions in sleep patterns, increased levels of stress hormones, and changes in circadian rhythms, all of which may contribute to an increased risk of SCD [33, 34]. However, work-related stress and job strain can also contribute to the development of chronic conditions like diabetes and hypertension, which can alter cardiac structure and function as well as the heart’s electrical activity. For these reasons, these conditions are also regarded as risk factors for SCD [35]. Furthermore, stress and job strain can result in unhealthy coping strategies such as alcoholism, smoking, and poor eating habits, all of which can raise the risk of SCD [36].

SCD prevention is still a significant problem in contemporary medicine. The measures for risk reduction are influenced by the variety of its physiopathology, both acquired and hereditary factors concur to the development of fatal ventricular arrhythmias [37]. To prevent SCD among workers, it is important to identify those who may be at increased risk and provide them with appropriate screening and interventions. Screening employees who have a family history of SCD or other cardiovascular diseases could be one tactic to consider, as these people may be more susceptible due to genetic factors [38]. First-level instrumental exams, such as an electrocardiogram (ECG), which can identify irregularities in the electrical activity of the heart, can be used for screening.

ECG is a widely medical test used to measure the electrical activity of the heart. It is also used as a first-level test in screening for heart disease because it is non-invasive, simple, and quick to perform, and the results are typically available within a few minutes. In addition, ECG has a high diagnostic value in various heart conditions, including arrhythmias, heart attacks, and other abnormalities. Lastly, another advantage of this test is its low cost. It also has weaknesses, however, as it reports only limited information on the function of the heart (electrical activity), but it does not provide any information about the structure of the heart or blood vessels.

Alternatively, specialized cardiology consultations can be performed during the health surveillance visit to evaluate workers’ cardiovascular health and identify any risk factors for SCD. Other strategies to prevent SCD among workers may include promoting healthy lifestyle behaviors, such as regular physical activity, healthy eating, and stress reduction techniques [39–41]. Employers can also implement workplace policies that promote work-life balance, limit exposure to long working hours and shift work, and provide resources for managing job strain and work-related stress. It is also important for the employer in cooperation with the competent physician to train their employees on the risks posed by the disease and how they can prevent them.

### Strengths and limitations

This research provides valuable insights into the occurrence of sudden cardiac death among workers, enhancing our knowledge about the connection between the work environment and sudden cardiac events. One of the main strengths of the article is that it provides information on the prevalence of a risk factor for sudden cardiac death, such as cardiac channelopathies. In addition to advancing scientific understanding, this feature can have practical repercussions, such as the establishment of occupational health programs by companies, which can be useful in enhancing the safety and well-being of employees. By identifying risk factors at an early stage, targeted preventive actions can be implemented to improve cardiovascular health management in occupational settings.

The main limitations are related to the English language filters used in the initial search and the retrospective nature of the study designs of all included articles. In addition, the limited number of results drives us to consider the under-explored status of the topic and the need for further investigation.

## Conclusions

The prevalence of sudden cardiac death (SCD) among workers is subject to variation influenced by a multitude of factors, encompassing age, gender, occupational role, and pre-existing medical conditions. Predominantly, employees subjected to either physical or mental stress, those leading a sedentary lifestyle, or those with pre-established cardiac pathologies may exhibit an elevated susceptibility to SCD. The mitigation of SCD prevalence can be achieved through the identification and rectification of occupational risk factors, along with the implementation of targeted screenings and intervention strategies. Furthermore, fostering a culture of health consciousness within the workplace is imperative.

## Data Availability

Not applicable.
